# Elevated levels of adiponectin associated with major adverse cardiovascular and cerebrovascular events and mortality risk in ischemic stroke

**DOI:** 10.1186/s12933-020-01096-3

**Published:** 2020-08-08

**Authors:** Wen-Jun Tu, Han-Cheng Qiu, Ya-Kun Liu, Qiang Liu, Xianwei Zeng, Jizong Zhao

**Affiliations:** 1 Department of Neurosurgery, Beijing Tiantan Hospital, Capital Medical University, No. 119, South Four Ring West Road, Beijing, 100070 People’s Republic of China; 2 Department of Neurosurgery, Qilu Hospital of Shandong University, No. 107 Wenhua West Road, Jinan, 250012 Shandong People’s Republic of China; 3 Institute of Radiation Medicine, China Academy of Medical Science & Peking Union Medical College, Tianjin, China; 4 China National Clinical Research Center for Neurological Diseases, Beijing, China; 5 Center of Stroke, Beijing Institute for Brain Disorders, Beijing, China; 6 Beijing Key Laboratory of Translational Medicine for Cerebrovascular Disease, Beijing, China

**Keywords:** Adiponectin, Ischemic stroke, Mortality, Major adverse cardiovascular and cerebrovascular events, Chinese

## Abstract

**Background:**

Adiponectin plays role in multiple metabolic pathways. Previous studies in cardiovascular disease evaluated the association between adiponectin and clinical outcomes, yielding conflicting results. The aim of this study was to investigate the association of adiponectin with major adverse cardiovascular and cerebrovascular events (MACCE) and mortality in Chinese patients with first-ever acute ischemic stroke (AIS).

**Methods:**

This was a prospective, multicenter cohort study. From September 2009 through October 2015, all patients with AIS from 3 stroke centers in Shandong were included. Serum levels of adiponectin at admission were tested. The prognostic role of adiponectin to predict the MACCE and mortality within 3 years was evaluated by multivariable-adjusted Cox proportional hazards models.

**Results:**

This study included 4274 patients (median age 68 years [interquartile ranges {IQR}: 61–76]; 53.2% men). There were 794 deaths and 899 MACCE events. Higher serum levels of adiponectin on admission were found in patients with MACCE events and nonsurvivors (P < 0.001 and P < 0.001). In multivariable models adjusted for factors that confirmed in the univariate model, elevated serum levels of adiponectin were associated with a higher risk of MACCE (Quartile[Q]4 *vs*. Q1, Hazard ratio[HR] = 4.95 [95% confidence interval {CI}: 3.03–7.06]) and mortality (Q4 *vs*. Q1, HR = 5.63 [95% CI 3.15–7.99]). Adiponectin improved the prognostic value of the National Institutes of Health Stroke Scale (NIHSS) to predict MACCE (combined areas under the curve [AUC], 0.76; 95% CI 0.68–0.88; P = 0.001) and mortality (0.78[0.69–0.91]; P < 0.01). Subgroups analysis indicated that the prognostic role of adiponectin was more pronounced in women and patients with high levels of N-terminal-pro B-type natriuretic peptide(NT-pro BNP) (P < 0.001 and P < 0.001).

**Conclusions:**

Elevated serum levels of adiponectin were associated with a higher risk of MACCE and mortality independent of traditional risk factors in ischemic stroke patients.

## Background

In China, stroke is one of the main causes of death and long-term disability [1]. The GBD 2016 Lifetime Risk of Stroke Collaborators reported that China had the highest estimated lifetime risk of stroke from the age of 25 years onward (39.3%; 95% uncertainty interval [UI], 37.5 to 41.1) while the global risk was approximately 25% [2]. Novel and useful biomarkers that can predict stroke prognosis early are of great significance for improving patient prognosis and alleviating medical burden.

Adiponectin, first identified in 1995, is a 244–amino acid collagen-like protein [3]. It is secreted by adipocytes and plays role in anti-inflammatory and insulin-sensitizing properties [3]. The dysregulation of adiponectin has been implicated in obesity, metabolic syndrome, type 2 diabetes, hypertension, and cardiovascular disease [4, 5]. Adiponectin signaling plays role in the brain functions (fatty acid oxidation, energy homeostasis, hippocampal neurogenesis, and synaptic plasticity) through its receptors, AdipoR1 and AdipoR2 [6, 7].

Previous vitro studies illustrated that adiponectin could be seen as a cardioprotective molecule [8, 9]. Hu et al. [10] showed that higher levels of adiponectin related to decreased risk of cardiovascular diseases and mortality in US women. On the contrary, positive relationships between adiponectin and mortality in many clinical conditions, such as kidney disease [11], heart failure (HF) [12], cardiovascular disease (CVD) [13] and general elderly cohorts [14] had been declared. This contradictory phenomenon is so-called adiponectin “paradox” [15]. The biology underlying this paradox is unknown.

The adiponectin “paradox” also presents among patients with stroke. One study showed that elevated serum adiponectin levels were associated with a high risk of ischemic stroke [16], while other studies reported that decreased adiponectin related to post-stroke depression [17] and cerebrovascular disease [18]. Furthermore, some studies reported that adiponectin serum levels were not associated with risk of stroke among different populations [19, 20]. Similarly, one study showed that low adiponectin plasma levels were independently associated with a high risk of 5-year mortality after first-ever AIS [21], while another study indicated that elevated adiponectin levels related to a high risk of recurrence events and mortality in patients with coronary heart disease [22]. The pathophysiological and prognostic role of adiponectin in CVD and cerebrovascular disease is still obscure [23].

The aim of this study was to investigate the association of adiponectin with major adverse cardiovascular and cerebrovascular events (MACCE) and mortality in Chinese patients with first-ever acute ischemic stroke (AIS).

## Patients and methods

### Patient population

This was a prospective, multicenter cohort study. From September 2009 through October 2015, all patients with first-ever AIS with symptom onset within 24 h from 3 stroke centers in Shandong were included. Diagnosis of AIS was confirmed according to the World Health Organization ICD-9 criteria and computed tomography (CT) and/or magnetic resonance imaging (MRI) were used to verification diagnosis [24]. Exclusion criteria were as following: (1) malignant tumor and/or metabolic syndrome(not included diabetes mellitus); (2) renal and/or liver insufficiency (renal insufficiency was confirmed according to creatinine level [men with a creatinine level ≥ 1.5 mg/dl and women ≥ 1.3 mg/dl]); (3) surgical operation in the past 3 months; (4) acute and/or chronic medical illness (sepsis, pneumonia, infection and neurological illness other than stroke); and (5) no informed consent, lost blood specimens and lost flow-up.

### Clinical variables and neuroimaging

At admission, demographical and clinical data were collected: age, sex, body mass index (BMI, defined as the body weight [kg]divided by the square of the body height [m^2^]) and vascular risk factors (hypertension, diabetes mellitus, atrial fibrillation, hyperlipoproteinemia, coronary heart disease and smoking habit). The information about previous CVD events and family history for stroke were also collected. Pre-stroke treatment (Aspirin, Clopidogrel, Anticoagulation, Statins and Renin-angiotensin system blockers) and acute treatment information were obtained. Stroke severity on admission was evaluated using the National Institutes of Health Stroke Scale (NIHSS) [25]. Stroke subtype and syndrome were assessed by TOAST (Trial of Org 10172 in Acute Stroke Treatment) criteria and the Oxfordshire Community Stroke Project, respectively [26]. MRI was performed in some patients and diffusion-weighted imaging (DWI) lesion volumes was calculated [26].

### End points and follow-up

For follow-up, we used structured telephone interviews performed by 12 trained medical students, blinded to blood levels. In our study, the primary end point in stroke patients was incident MACCE (a composite of CVD death, nonfatal myocardial infarction, nonfatal stroke, or coronary revascularization by percutaneous coronary intervention or coronary artery bypass grafting) [27] of stroke patients after 3 years from baseline. Secondary end point was all-cause mortality within a 3-year follow-up.

### Blood samples test

For all patients, fasting serum samples were collected within 24 h of admission and stored at −80 °C. The serum concentration of adiponectin was tested by an enzyme-linked immunosorbent assay (ELISA) method (No, ab99968; Abcam Trading [Shanghai] Company Ltd. Shanghai, China) with a detection limit of 0.25 μg/mL. The intra-assay and inter-assay coefficients of variation (CV) were all less than 10.0%. Other biomarkers including glucose, C-reactive protein (CRP), copeptin and N-terminal of the prohormone B-type natriuretic peptide (NT-proBNP) were also tested [24]. In addition, the estimated glomerular filtration rate (eGFR) of these subjects was calculated according to the creatinine levels using the Chronic Kidney Disease Epidemiology Collaboration (CKD-EPI) formula [28]. Two hundred age and gender-matched healthy volunteers from our medical center were chosen to assess the normal concentration range of serum adiponectin. Volunteers with any sub-clinical stroke features would be excluded. The fasting serum samples of those volunteers were collected and serum levels of adiponectin were tested.

### Statistical analysis

In this study, categorical variables were presented as number (percentages,  %) and continuous variables were presented as medians (interquartile ranges, IQRs). Continuous variables were tested for normality by Shapiro–Wilk tests. Mann–Whitney U test (continuous variables) or Chi square test (categorical variables) was used for comparison between groups. Spearman’s rank was used to assess the bivariate correlations.

The association between adiponectin and two endpoints (MACCE and mortality) was evaluated by the Cox proportional hazards models and the results were reported as hazard ratios (HR) and 95% confidence interval (CI). Multivariable models were adjusted for those significantly factors which had been confirmed in the univariate model.

The accuracy of serum adiponectin in predicting wo endpoints (MACCE and mortality) was analyzed by receiver operating characteristic (ROC) curves and the results were reported as area under the curve (AUC) and 95%CI. The cut-off of adiponectin value was calculated. The clinical value of adding adiponectin to the existing risk factors for predicting the two endpoints was further calculated by the net reclassification improvement (NRI) index. In addition, to investigate the ability of adiponectin for wo endpoints (MACCE and mortality) prediction, we used Kaplan–Meier survival curves and stratified patients by adiponectin quartiles and compared with the log-rank test.

Lastly, we performed subgroup analyses and stratified patients by age (< 60 vs. ≥ 60 years), sex (men vs. women), BMI (< 27 vs. ≥ 27 kg/m^2^), diabetes status (yes vs. no), eFGR(< 60 vs. ≥ 60 mL/min/1.73 m^2^), CRP(< 5 vs. ≥ 5 mg/l), copeptin (< median vs. ≥ median)and NT-ProBNP(< median vs. ≥ median). We used the following statistical software: SPSS Statistics (version 24.0; IBM Corp., Armonk, NY, USA), the ROCR package (version 1.0–2; http://cran.r-project.org/) and GraphPad Prism (version 5.0; GraphPad Software, La Jolla, CA, USA). A P value < 0.05 (2-sided) indicated significance.

## Ethics

The design and proposal were reviewed and approved by Ethics Committee of the Weifang Medical University Affiliated Hospital according to the principles of the Declaration of Helsinki (trial registration no. ChiCTR-ROC-17013501). Written informed consents were collected from patients or their relatives (patients unable to communicate) before they participated in the study.

## Results

### Patient characteristics

**Fig. 1:**
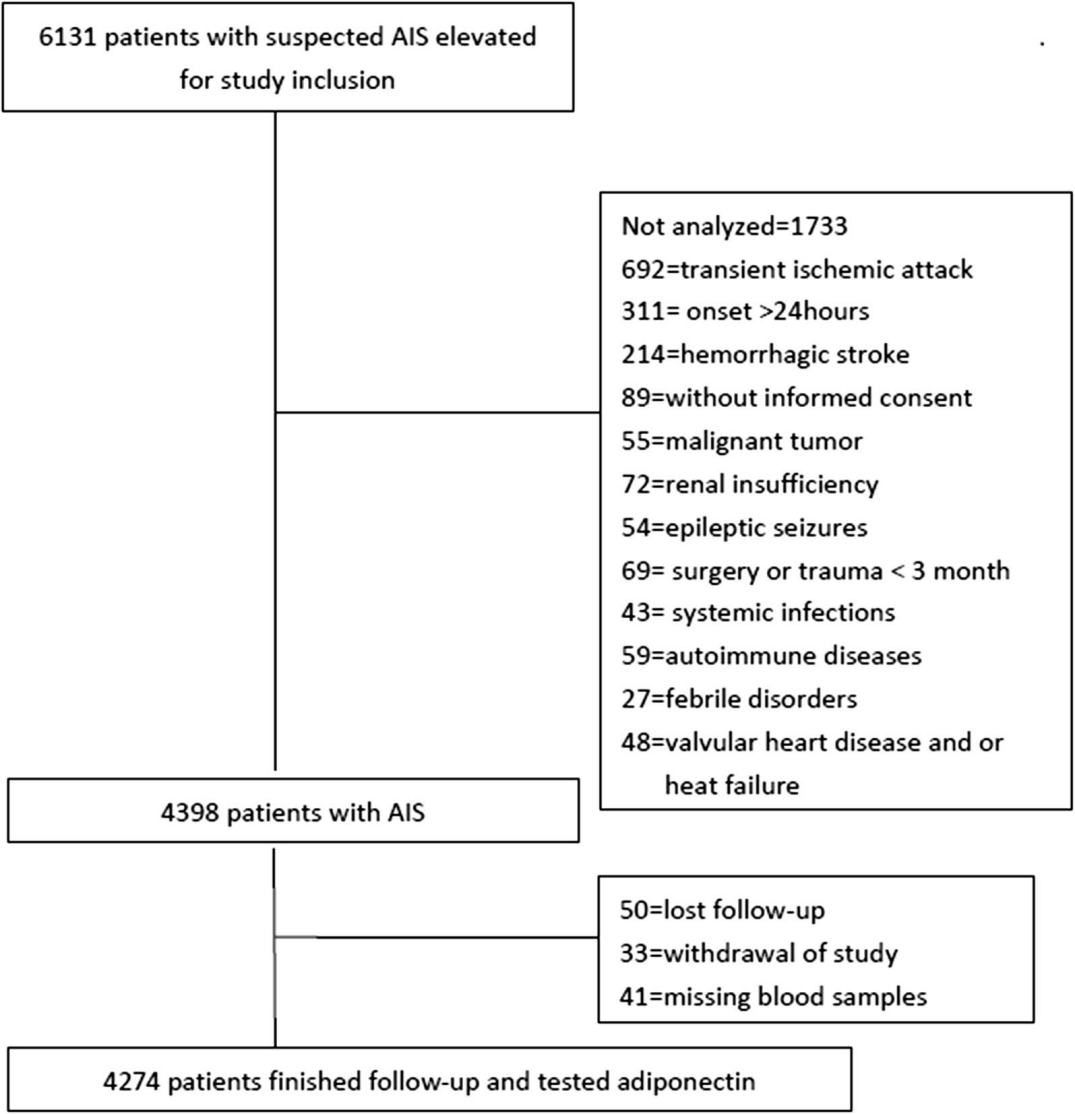
Study profile/flow sheet of the study

Finally, as shown in the Fig. 1, 4274 patients with AIS were included. The baseline characteristics [age (P = 0.62), gender (P = 0.87), and BMI (P = 0.81)] of those patients were similar to the overall cohort. The median follow-up period was 3.0 years (mean: 3.03 years). In addition, 53.2% of those patients were male and the median age of was 68 years (IQR:61–76). The median serum level of adiponectin at admission was 7.1 μg/mL (IQR:4.8–9.8), which was higher than in those healthy controls (6.0[4.1–7.2] μg/mL), *P *< 0.001. More information had been presented in the Table 1.

**Table 1:** Baseline characteristics of stroke patients^a^

Baseline characteristics	All
N	4274
Age, years	68 (61–76)
Male gender	2274 (53.2)
BMI, kg/m2	26.2 (24.4–28.6)
Vascular risk factors	
Hypertension	1848 (43.2)
Diabetes mellitus	1011 (23.7)
Hypercholesterolemia	881 (20.6)
Atrial fibrillation	842 (19.7)
Coronary heart disease	1103 (25.8)
Current smoking	762 (17.8)
Previous CVD events	791 (18.5)
Family history for stroke	693 (16.2)
NIHSS score at admission	7 (4–13)
DWI lesion size (N = 2812), ml	21.3 (7.2–41.8)
Time to blood collection, hours	5.2 (3.2–11.1)
Stroke syndrome	
TACS	966 (22.6)
PACS	1650 (38.6)
LACS	752 (17.6)
POCS	906 (21.2)
TOAST subtype	
Large-vessel disease	756 (17.7)
Small-artery disease	700 (16.4)
Cardioembolic	1041 (24.4)
Multiple causes	758 (17.7)
Other known	558 (13.1)
Undetermined	461 (10.8)
Laboratory findings, serum levels	
Glucose, mmol/l	6.3 (5.5–7.5)
Creatinine, mmol/l	81.1 (69.0–95.0)
eGFR, e mL/min/1.73 m^2^	75.0 (60.8–91.9)
CRP, mg/l	3.9 (2.4–9.5)
Copepin, pmol/l	20.1 (14.9–28.3)
NT-ProBNP, pg/ml	1111 (266–3722)
Adiponectin, μg/mL	7.1 (4.8–9.8)
Therapies before admission	
Aspirin	900 (21.1)
Clopidogrel	464 (10.9)
Anticoagulation	855 (20.0)
Statins	1302 (30.5)
Renin-angiotensin system blockers	1541 (36.1)
Acute treatment	
Endovascular/surgical revascularization	312 (7.3)

*BMI* body mass index, *NIHSS* National Institutes of Health Stroke Scale, *TACS* total anterior circulation syndrome, *PACS* partial anterior circulation syndrome, *LACS* lacunar syndrome, *POCS* posterior circulation syndrome, *GFR* glomerular filtration rate, *CRP* C–reactive protein, *NT-proBNP* N-terminal fragment of precursor of B-type natriuretic peptide, *CVD* cardiovascular disease

^a^The results were presented as n(percentages) for categorical variables and as medians (interquartile ranges,IQRs) for continuous variables

### Baseline adiponectin and MACCE

**Fig. 2:**
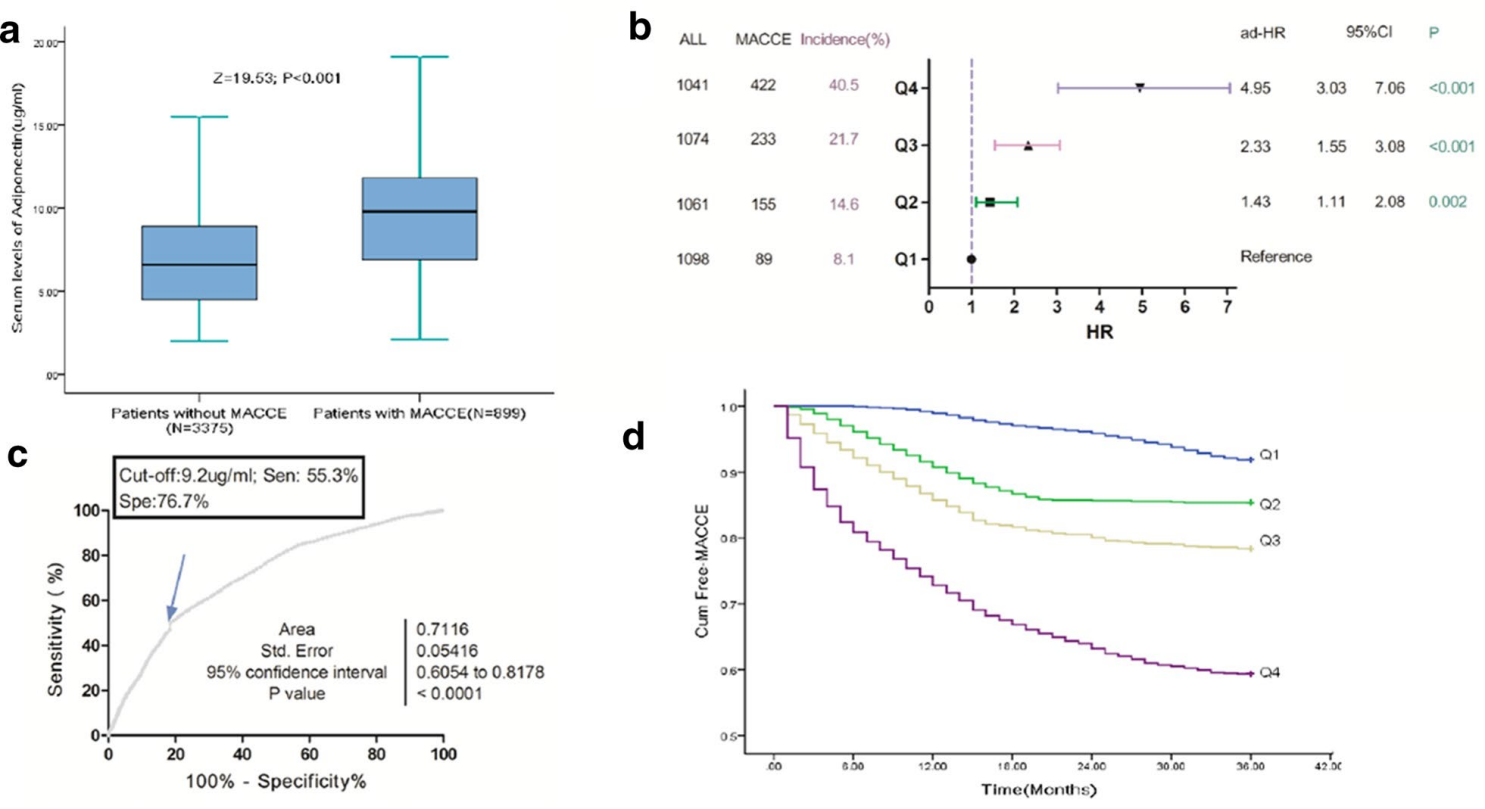
Baseline serum levels of adiponectin and MACCE at 3-year follow-up. **a** Serum levels of adiponectin in patients with MACCE and free-MACCE. **b** Multivariate analyses for MACCE according to adiponectin quartiles. Adjustments for age, sex (female vs. male), obese (yes vs. no), diabetes mellitus (yes vs. no), atrial fibrillation (yes vs. no), previous CVD events (yes vs. no), NHISS, stroke syndrome (TACS vs. other), stroke subtype (Cardioembolic vs. other), acute treatment (no vs. yes), serum levels of glucose, CRP, eGFR, copeptin and NT-ProBNP. **c** ROC curves were utilized to evaluate the accuracy of serum level of adiponectin to predict MACCE. **d** The Kaplan–Meier estimates of MACCE stratified by baseline adiponectin quartiles. Adiponectin quartiles were defined as Q1 < 4.8 μg/mL, Q2 4.8–7.1 μg/mL, Q3 7.2–9.8 μg/mL and Q4 > 9.8 μg/mL. MACCE was defined as CVD death, nonfatal myocardial infarction, nonfatal stroke, or coronary revascularization by percutaneous coronary intervention or coronary artery bypass grafting. *HR* Hazard ratio, *CI* confidence interval, *BMI* body mass index, *NIHSS* National Institutes of Health Stroke Scale, *CRP* C-reactive protein, *NT-proBNP* N-terminal fragment of precursor of B-type natriuretic peptide, *TACS* total anterior circulation syndrome, *GFR* glomerular filtration rate, *CVD* cardiovascular disease, *MACCE* major adverse cardiovascular and cerebrovascular events

During the 3-year follow-up period, 899 patients (21.0%; 95%CI 19.8%–22.3%) were defined as MACCE, and the adiponectin serum levels in those patients were significantly higher than those without MACCE (9.8[IQR: 6.9–11.8] μg/mL vs. 6.6[4.5–8.9] μg/mL; *P *< 0.001), Fig. 2a. Elevated serum levels of adiponectin (continuous variable) related to a high risk of MACCE. Per one-unit (μg/mL) increase, the risk of MACCE increased by 20% (HR, 1.20; 95%CI 1.1–8-1.23), Table 2. The risk of MACCE was distributed across adiponectin quartiles, from 8.1% (first quartile, Q1) to 40.5% (fourth quartile, Q4). Patients in the Q4 had a 6.73-fold higher risk of MACCE (HR, 7.73; 95%CI 6.02–9.92) as compared with patients in Q1. Similarly, patients in the 2nd and 3rd quartiles of adiponectin also related to high risk of MACCE and the risk increased by 94% (HR, 1.94;95%CI 1.47–2.56) and 214% (3.14; 2.42–4.08), respectively.

**Table 2:** Univariate Cox regression analysis for MACCE and Mortality in stroke patients during 3-year follow-up

Parameter	MACCE^a^			Mortality		
	HR	95% CI	*P*	HR	95% CI	*P*
Age (increase per unit)	1.08	1.02–1.16	0.005	1.13	1.06–1.19	< 0.001
Sex (female *vs.* male)	1.65	1.19–2.55	0.015	1.33	0.85–2.18	0.39
BMI (≥ 30 vs. < 30 kg/m^2^)	1.21	1.07–1.37	0.009	1.28	1.08–1.43	0.005
Vascular risk factors (yes vs. no)						
Hypertension	1.63	1.05–3.01	0.29	1.75	0.93–3.15	0.21
Diabetes mellitus	1.25	1.07–1.46	0.012	1.09	0.85–2.41	0.59
Atrial fibrillation	1.81	1.28–2.74	0.007	3.01	0.95–5.33	0.17
Coronary heart disease	1.48	0.90–2.77	0.42	2.11	1.02–3.43	0.07
Hypercholesterolemia	0.85	0.73–1.54	0.27	0.93	0.82–1.42	0.19
Current smoking	1.43	0.89–2.29	0.39	1.76	0.73–3.88	0.53
Previous CVD events	1.25	1.09–1.49	0.009	1.32	1.02–1.74	0.011
Family history for stroke	0.89	0.69–1.59	0.59	0.93	0.73–2.05	0.52
Infarct volume (increase per unit), n = 2810	1.25	1.12–1.39	0.007	1.31	1.10–1.44	0.006
NIHSS (increase per unit)	1.17	1.12–1.23	< 0.001	1.19	1.14–1.27	< 0.001
Time to blood collection	1.59	0.83–2.74	0.45	1.79	0.93–3.15	0.63
Stroke syndrome						
TACS	3.75	1.85–6.65	0.001	4.72	2.03–7.59	< 0.001
PACS	1.55	0.75–2.65	0.61	0.85	0.41–1.66	0.61
LACS	0.70	0.44–1.63	0.39	0.59	0.36–1.32	0.13
POCS	0.43	0.25–0.65	0.015	0.48	0.23–1.03	0.20
TOAST subtype						
Large-vessel disease	0.95	0.55–1.98	0.43	0.55	0.32–1.32	0.26
Small-artery disease	0.76	0.47–1.75	0.38	0.69	0.39–1.54	0.33
Cardioembolic	1.19	1.02–1.62	0.032	1.55	1.08–2.03	0.016
Multiple causes	0.93	0.66–1.76	0.64	0.82	0.45–2.04	0.87
Other known	1.51	0.99–2.14	0.09	1.42	0.87–2.01	0.18
Undetermined	0.65	0.21–1.61	0.28	0.72	0.33–1.82	0.31
Therapies before admission						
Aspirin	0.95	0.46–2.38	0.85	0.93	0.76–2.87	0.93
Clopidogrel	1.74	0.77–3.92	0.68	1.55	0.80–3.05	0.51
Anticoagulation	0.83	0.45–1.79	0.07	0.76	0.54–1.56	0.15
Statins	1.25	0.67–1.97	0.56	1.22	0.98–1.65	0.09
Renin-angiotensin system blockers	0.93	0.80–1.48	0.23	0.90	0.69–1.65	0.18
Acute treatment (no vs. yes)	3.78	2.04–5.54	< 0.001	4.48	3.01–6.12	< 0.001
Blood biomarkers						
Glucose (increase per unit)	1.09	1.01–1.27	0.031	1.12	1.03–1.22	0.003
Creatinine (increase per unit)	1.02	0.90-1.48	0.27	1.05	0.85-1.53	0.31
eGFR (increase per unit)	1.05	1.01–1.12	0.011	1.09	1.03–1.24	0.028
CRP (increase per unit)	1.05	1.01–1.10	0.006	1.09	1.02–1.19	0.013
Copepin (Q4 vs. Q1-3)	3.74	2.55–5.39	< 0.001	4.83	3.04–7.16	< 0.001
NT-ProBNP (Q4 vs. Q1-3)	3.15	2.12–4.55	< 0.001	3.98	2.36–5.47	< 0.001
Adiponectin (increase per unit)	1.20	1.18–1.23	< 0.001	1.23	1.20–1.27	< 0.001

*HR* Hazard ratio, *CI* confidence interval, *BMI* body mass index, *NIHSS* National Institutes of Health Stroke Scale, *CRP* C-reactive protein, *NT-proBNP* N-terminal fragment of precursor of B-type natriuretic peptide, *TACS* total anterior circulation syndrome, *GFR* glomerular filtration rate, *CVD* cardiovascular disease, *MACCE* major adverse cardiovascular and cerebrovascular events

^a^MACCE was defined as CVD death, nonfatal myocardial infarction, nonfatal stroke, or coronary revascularization by percutaneous coronary intervention or coronary artery bypass grafting

As showed in the Table 2, univariate Cox regression analysis for MACCE were performed. We further evaluated potentially factors: sex, BMI, NTpro-BNP, and CRP. Women patients had higher levels of adiponectin when compared with men patients (7.9[IQR: 5.8-10.7] μg/mL vs. 6.6[4.2–9.0] μg/mL; p < 0.001). Adiponectin was inversely correlated to BMI (r = 0.198, P < 0.001), positively to NT-pro-BNP (r = 0.275, P < 0.001) and CRP (r = 0.216, P < 0.001). Adjustments for age, sex (female vs. male), obese (yes vs. no), diabetes mellitus (yes vs. no), atrial fibrillation (yes vs. no), previous CVD events (yes vs. no), NHISS, stroke syndrome (TACS vs. other), stroke subtype (Cardioembolic vs. other), acute treatment (no vs. yes), serum glucose, CRP, eGFR, copeptin and NT-ProBNP, per one-unit(μg/mL) increase in adiponectin, the risk of MACCE increased by 13% (HR, 1.13; 95%CI 1.06–1.19), Table 3. In the analysis of multivariable models, the Q2, Q3, and Q4 of adiponectin levels were compared against Q1 (Fig. 2b). As shown in the Fig. 2b, adiponectin levels in Q2, Q3, and Q4 related to MACCE and the risk was increased by 43% (HR 1.43, 95% CI 1.11–2.08), 133% (2.33, 1.55–3.08) and 395% (4.95, 3.03–7.06), respectively as compared with patients in Q1. The independence of correlation between adiponectin and MACCE was tested by the likelihood ratio test (P < 0.001). In addition, age, BMI, diabetes mellitus, previous CVD events, acute treatment, CRP, copeptin, BNP and the NIHSS score were significant MACCE predictors, unlike others factors (Table 3).

**Table 3:** Multivariate Cox analysis of MACCE and mortality in stroke patients during 3-year follow-up

Variable	MACCE^a^		Mortality^b^	
	HR (95%CI)	P	HR (95%CI)	P
Age (increase per unit)	1.08 (1.01–1.16)	0.009	1.11 (1.04–1.19)	0.006
Sex (female vs. male)	1.43 (0.90–2.15)	0.16	–	
BMI (≥ 30 *vs.* < 30 kg/m2)	1.16 (1.04–1.26)	0.018	1.18 (1.03–1.29)	0.016
Diabetes mellitus (yes vs. no)	1.11 (1.02–1.32)	0.042	–	
Atrial fibrillation (yes vs. no)	1.48 (0.92–2.67)	0.063	–	
Previous CVD events (yes vs. no)	1.12 (1.02–1.25)	0.016	1.15 (0.99–1.38)	0.15
NIHSS (increase per unit)	1.14 (1.07–1.23)	< 0.001	1.16 (1.09–1.24)	< 0.001
Stroke syndrome (TACS vs. other)	2.25 (0.75–4.32)	0.38	2.56 (0.68–4.76)	0.43
Stroke subtype (Cardioembolic vs. other)	1.07 (0.86–2.14)	0.26	1.14 (0.97–1.89)	0.18
Acute treatment (no vs. yes)	3.01 (1.98–4.05)	< 0.001	3.75 (2.96–4.49)	< 0.001
Glucose (increase per unit)	1.07 (0.98–1.25)	0.15	1.09 (1.00–1.23)	0.076
eGFR (increase per unit)	1.02 (0.93–1.43)	0.13	1.03 (1.00–1.11)	0.045
CRP (increase per unit)	1.03 (1.00–1.09)	0.015	1.05 (1.01–1.11)	0.011
Copeptin (Q4 vs. Q1-3)	2.98 (1.55–4.07)	0.001	3.65 (2.12–5.05)	< 0.001
NT-pro BNP (Q4 vs. Q1-3)	2.05 (1.21–3.39)	0.009	3.03 (1.87–4.23)	0.003
Adiponectin (increase per unit)	1.13 (1.06–1.19)	< 0.001	1.17 (1.11–1.24)	< 0.001

*HR* Hazard ratio, *CI* confidence interval, *BMI* body mass index, *NIHSS* National Institutes of Health Stroke Scale, *CRP* C-reactive protein, *NT-proBNP* N-terminal fragment of precursor of B-type natriuretic peptide, *TACS* total anterior circulation syndrome, *GFR* glomerular filtration rate, *CVD* cardiovascular disease, *MACCE* major adverse cardiovascular and cerebrovascular events

^a^Adjusted for age, sex (female vs. male), BMI (yes vs. no), Diabetes mellitus (yes vs. no), Atrial fibrillation (yes vs. no), Previous CVD events (yes vs. no), NHISS, Stroke syndrome (TACS vs. other), Stroke subtype (Cardioembolic vs. other), Acute treatment (no vs. yes), serum levels of Glucose, CRP, eGFR, Copeptin, NT-ProBNP and Adiponectin. MACCE was defined as CVD death, nonfatal myocardial infarction, nonfatal stroke, or coronary revascularization by percutaneous coronary intervention or coronary artery bypass grafting

^b^Adjusted for age, BMI (yes vs. no), Previous CVD events (yes vs. no), NHISS, Stroke syndrome (TACS vs. other), Stroke subtype (Cardioembolic vs. other), Acute treatment (no vs. yes), serum levels of Glucose, CRP, eGFR, Copeptin, NT-ProBNP and Adiponectin

The ROC curve was applied for choosing the cut-off value of adiponectin in predicting MACCE, Fig. 2c. An optimal value of 9.2 μg/mL produced a sensitivity of 55.3% and a specificity of 76.7%, with AUC (95% CI) of 0.71 (0.61–0.82), which showed a significantly greater discriminatory ability as compared with other factors (Table 4). There was a positive association with MACCE when adiponectin levels above cut-off point (HR 3.21, 95% CI 2.36–4.14; P < 0.001).

**Table 4:** Area under the curve for selected predictors of MACCE and mortality

Predictors	MACCE^a^		Mortality	
	AUC (95%CI)	P	AUC (95%CI)	P
Adiponectin	0.71 (0.61–0.82)	–	0.73 (0.58-0.89)	–
Age	0.63 (0.55–0.72)	< 0.001	0.65 (0.56 –0.74)	< 0.001
CRP	0.65 (0.57 –0.75)	< 0.001	0.68 (0.59 –0.77)	< 0.001
NIHSS	0.73 (0.65 –0.84)	0.37	0.75 (0.66 –0.90)	0.42
Copeptin	0.73 (0.66 –0.85)	0.17	0.74 (0.64 –0.88)	0.28
NT-proBNP	0.68 (0.58 –0.77)	0.009	0.70 (0.58 –0.85)	0.011
Model I^b^	0.76 (0.68 –0.88)	0.001	0.78 (0.69 –0.91)	< 0.001
Model II^c^	0.79 (0.70 –0.90)	< 0.001	0.82 (0.71 –0.94)	< 0.001
Model III^d^	0.77 (0.68 –0.86)	< 0.001	0.80 (0.69 –0.92)	< 0.001
Model IV^e^	0.81 (0.69 –0.93)	0.013^f^	0.84 (0.72 –0.96)	0.005^f^

*MACCE* major adverse cardiovascular and cerebrovascular events, *CI* confidence interval, *AUC* Area Under Curve, *NIHSS* National Institutes of Health Stroke Scale, *CRP* C-reactive protein, *NT-proBNP* N-terminal fragment of precursor of B-type natriuretic peptide

^a^MACCE was defined as CVD death, nonfatal myocardial infarction, nonfatal stroke, or coronary revascularization by percutaneous coronary intervention or coronary artery bypass grafting

^b^Model I included NIHSS and Adiponectin

^c^Model II included NIHSS, Adiponectin, Copeptin and NT-proBNP

^d^Model III included NIHSS, CRP, age, Copeptin and NT-proBNP

^e^Model IV included model III and Adiponectin

^f^The P value was compared with Model III

In a combined model, the AUC of the NIHSS score could be increased with adiponectin (0.76; 95% CI 0.68–0.88, P = 0.001). As shown in the Table 4, the combined model IV (with adiponectin) improved the model III (without adiponectin) (AUC, 0.81[0.69–0.93] vs. 0.77[0.68–0.86]; P = 0.013). The average AUC (standard error) for combined IV and III were 0.81(0.025) and 0.77 (0.036), corresponding to a difference of 0.04 (0.011). The inclusion of adiponectin as a variable showed a significant improvement in risk estimation over traditional variables for MACCE at 3 years as monitored by NRI (0.32; 95%CI [0.25–0.41]; P < 0.001). The Kaplan–Meier estimates of MACCE stratified by baseline adiponectin quartiles was showed in Fig. 2d. Patients in the lowest quartile (adiponectin < 4.8 μg/mL) had a minimal risk for MACCE, in contrast with patients with adiponectin levels in the 2nd, 3rd and 4th quartiles (adiponectin between 4.8 and 7.1 μg/mL, 7.1 and 9.8 μg/mL and  > 9.8 μg/mL, respectively) (P < 0.001, log-rank test).

### Baseline adiponectin and all-cause mortality

**Fig. 3:**
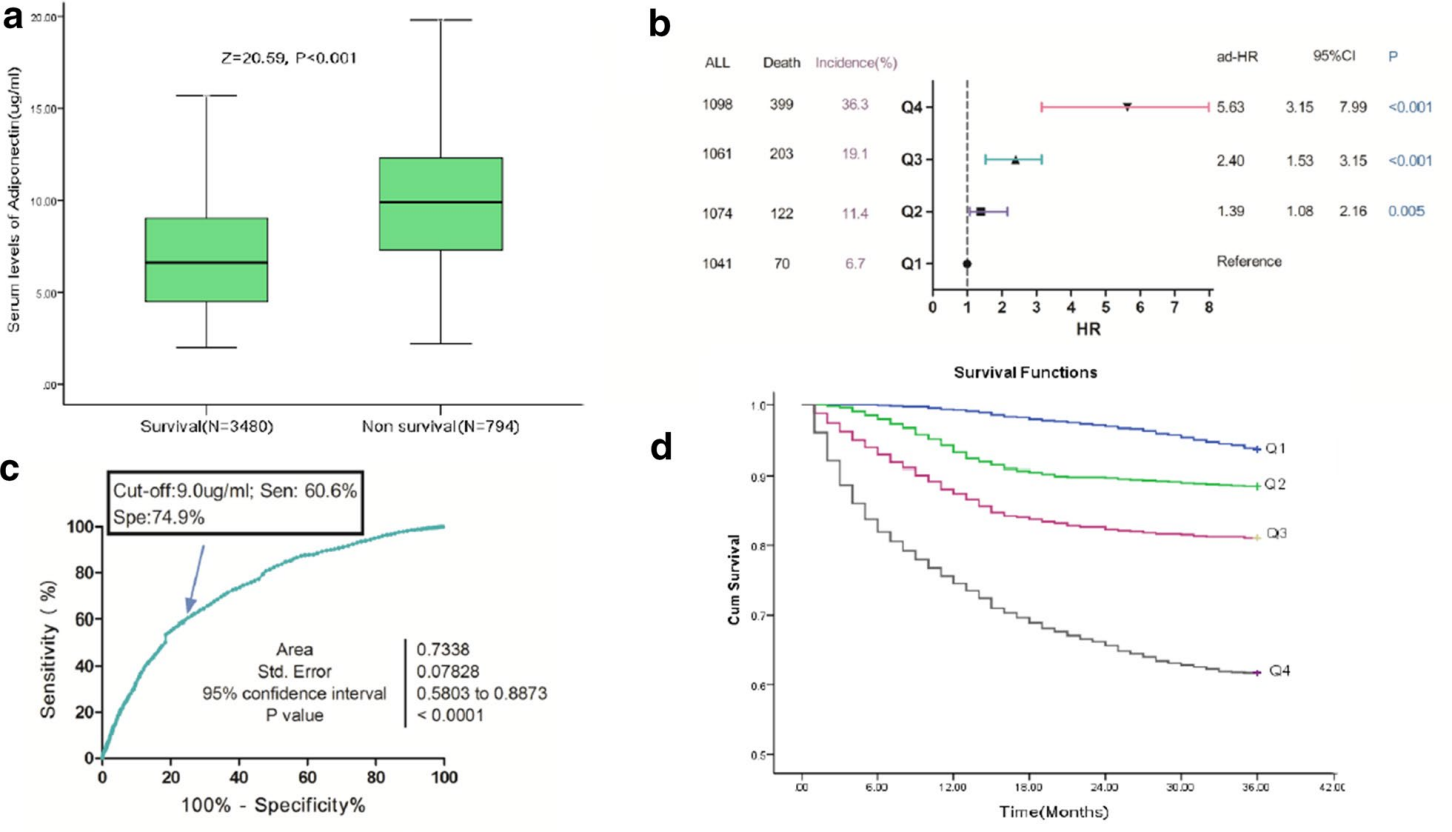
Baseline serum levels of adiponectin and morality at 3-year follow-up. **a** Serum levels of adiponectin in survival and non-survival. **b** Multivariate analyses for mortality according to adiponectin quartiles. Adjustments for age, obese (yes vs. no), previous CVD events (yes vs. no), NHISS, stroke syndrome (TACS vs. other), stroke subtype (Cardioembolic vs. other), acute treatment (no vs. yes), serum levels of glucose, CRP, eGFR, copeptin and NT-ProBNP. **c** ROC curves were utilized to evaluate the accuracy of serum level of adiponectin to predict mortality. **d** The Kaplan–Meier estimates of mortality stratified by baseline adiponectin quartiles. Adiponectin quartiles were defined as Q1 < 4.8 μg/mL, Q2 4.8–7.1 μg/mL, Q3 7.2–9.8 μg/mL and Q4 > 9.8 μg/mL. MACCE was defined as CVD death, nonfatal myocardial infarction, nonfatal stroke, or coronary revascularization by percutaneous coronary intervention or coronary artery bypass grafting. Abbreviation see the Fig. 2

Finally, 794 patients (18.6%; 95%CI 17.5%–19.9%) died, and serum adiponectin levels in those patients were higher than in those survivals (9.9[IQR: 7.3–12.3] μg/mL vs. 6.6[4.5–9.0] μg/mL; P < 0.001), Fig. 3a. Elevated serum adiponectin related to a high risk of mortality. Per one-unit(μg/mL) increase in adiponectin, the risk of mortality increased by 23% (HR, 1.23; 95%CI 1.20–1.27), Table 2. The risk of mortality was distributed across adiponectin quartiles, from 6.7% (first quartile, Q1) to 36.3% (fourth quartile, Q4). Patients in the 2nd, 3rd and highest quartiles of adiponectin had higher risk of mortality and the risk was increased by 91% (HR, 1.91;95%CI 1.30–2.59), 242% (3.42; 2.57–4.56) and 813% (9.13; 6.95–11.99), respectively as compared with patients in the Q1.

As showed in the Table 2, univariate Cox regression analysis for mortality were performed. Adjustments for age, obese (yes vs. no), previous CVD events (yes vs. no), NHISS, stroke syndrome (TACS vs. other), stroke subtype (Cardioembolic vs. other), acute treatment (no vs. yes), serum levels of glucose, CRP, eGFR, copeptin and NT-ProBNP, per one-unit(μg/mL) increase in adiponectin, the risk of mortality increased by 17% (HR, 1.17; 95%CI 1.11–1.24), Table 3. In the analysis of multivariable models, the 2nd, 3rd and highest adiponectin quartiles levels were compared against lowest quartile (Fig. 3b). As shown in the Fig. 3b, adiponectin levels in Q2, Q3, and Q4 were correlated with mortality and the risk was increased by 39% (HR 1.39, 95% CI 1.08–1.26), 140% (2.40, 1.53–3.15) and 463% (5.63, 3.15–7.99), respectively. The independence of correlation between adiponectin and mortality was tested by the likelihood ratio test (P < 0.001). In addition, age, BMI, acute treatment, CRP, copeptin, BNP and the NIHSS score were significant mortality predictors, unlike others factors (Table 3).

The ROC curve was applied for choosing the cut-off value of adiponectin in predicting MACCE, Fig. 3c. An optimal value of 9.0 μg/mL produced a sensitivity of 60.6% and a specificity of 74.9%, with AUC (95% CI) of 0.73 (0.58–0.89), which showed a significantly greater discriminatory ability as compared with other factors (Table 4). There was a positive association with total mortality when adiponectin levels above cut-off point (HR 4.45, 95% CI 3.28–5.81; P < 0.001).

In a combined model, the AUC of the NIHSS score could be increased with adiponectin (0.78; 95% CI 0.69–0.91, P < 0.001). As shown in the Table 4, the combined model IV (with adiponectin) improved the combined model III (without adiponectin) (AUC [95%CI], 0.84[0.72–0.96] *vs.* 0.80[0.69–0.92]*; P *= *0.005).* The average AUC (standard error) for combined IV and III were 0.84(0.015) and 0.80 (0.026), corresponding to a difference of 0.04 (0.011). The inclusion of adiponectin as a variable showed a significant improvement in risk estimation over traditional variables for mortality at 3 years as monitored by NRI (0.35; 95%CI 0.27–0.45; P < 0.001). The Kaplan–Meier estimates of mortality stratified by baseline adiponectin quartiles was showed in Fig. 3d. Again, patients in the lowest quartile (adiponectin < 4.8 μg/mL) had a minimal risk for mortality, in contrast with patients with adiponectin levels in the 2nd, 3rd and 4th quartiles (adiponectin between 4.8 and 7.1 μg/mL, 7.1 and 9.8 μg/mL and > 9.8 μg/mL, respectively) (P < 0.001, log-rank test).

### Subgroup analysis

**Fig. 4:**
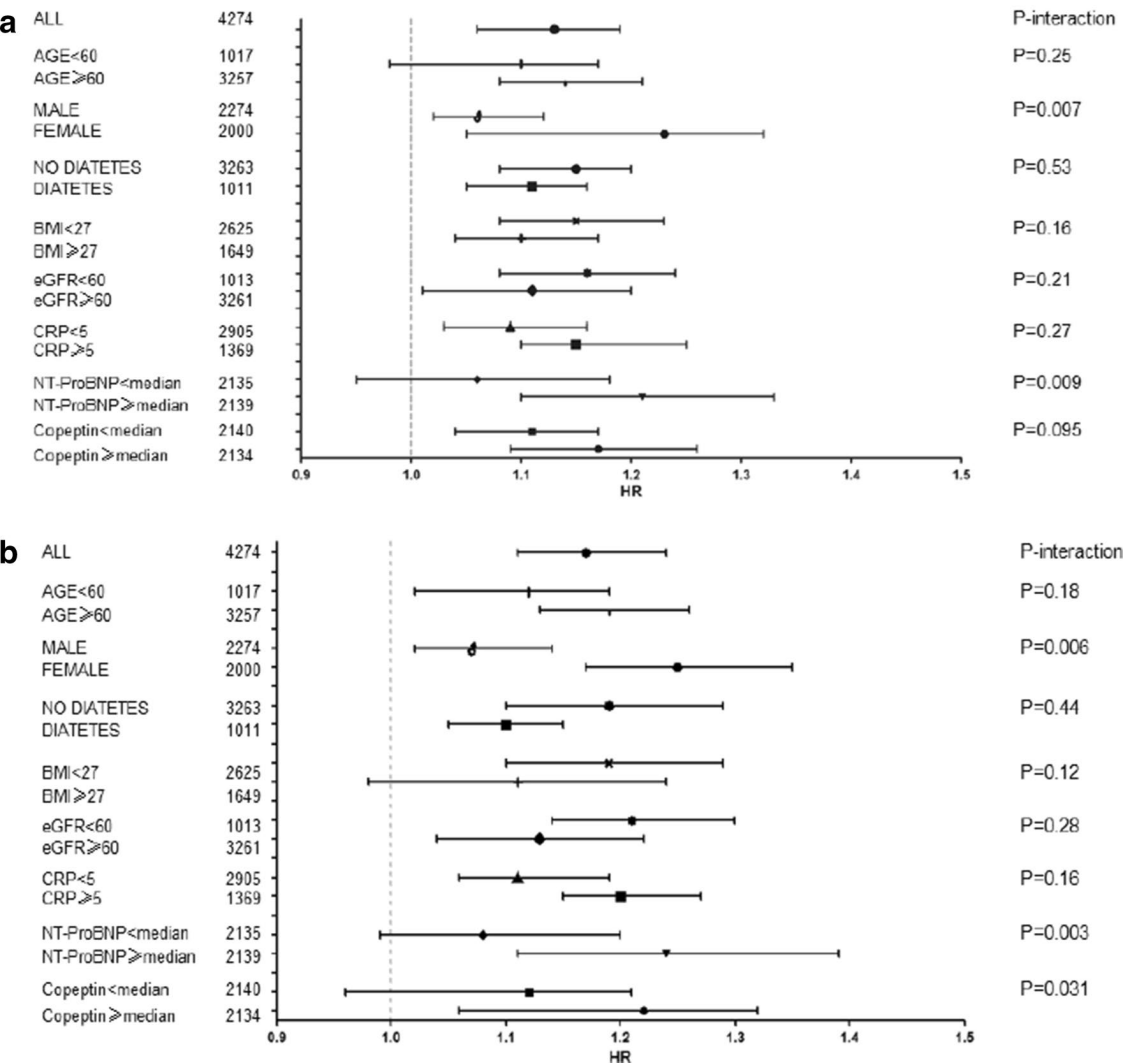
Hazard ratio with 95% CI for the association of adiponectin level with MACCE and all-cause mortality stratified by subgroups. **a** Hazard ratio with 95% confidence intervals for the association of adiponectin level with MACCE stratified by subgroups. **b** Hazard ratio with 95% confidence intervals for the association of adiponectin level with all-cause mortality stratified by subgroups. Abbreviation see the Fig. 2

HR with 95% CI for the association of adiponectin level with MACCE and all-cause mortality stratified by subgroups (Fig. 4). As shown in the Fig. 4a, NT-pro-BNP and female enhanced the association of adiponectin with MACCE. Interestingly, the predictive value of adiponectin to predict MACCE was stronger in women patients than in those men patients (HR, 1.23; 95%CI 1.15–1.32; *vs.* 1.06, 1.02–1.12; P = 0.007). Similarly, adiponectin in those patients with higher levels of NT-ProBNP also presented better predictive value (P = 0.009). As shown in the Fig. 4b, the predictive value of adiponectin to predict mortality was stronger in women patients and those patients had higher levels of NT-ProBNP and copeptin (P < 0.05, all).

## Discussion

Adiponectin plays role in cerebral nervous system [29]. Adiponectin exhibits distinct associations with mortality in elders, and the adiponectin paradox as it relates to older adults had been proposed [30]. In addition, Kuwashiro et al. [31] reported that plasma levels of adiponectin were associated with neurological severity and prognosis in patients with ischemic stroke patients. This study was the first population-based study on the correlation between circulating levels of adiponectin and risk of MACCE and mortality in Chinese patients with AIS. The main findings were as following: (1) higher serum levels of adiponectin on admission were found in patients with MACCE events and nonsurvivors; (2) in multivariable models, elevated serum levels of adiponectin related to a high risk of MACCE (Q4 vs. Q1, HR = 4.95, 95% CI 3.03–7.06) and mortality (Q4 vs. Q1, HR = 5.63, 95% CI 3.15–7.99); (3) the prognostic accuracy of the NIHSS to predict MACCE (P = 0.001) and mortality (P < 0.001) was improved by adiponectin; (4) the predictive value of adiponectin was more pronounced in women and patients with high levels of NT-pro-BNP (P < 0.001, all).

In fact, a paradoxical association between adiponectin and CVD events and death had been presented in previous studies. One study reported that high plasma adiponectin related to mortality during the 17 months follow-up in patients with acute stroke [32], while another study showed that higher leptin/adiponectin ratio at day 1 related to better neurological outcomes in atherothrombotic AIS [33]. Wang et al. [34] reported that high adiponectin could be seen as a prognostic marker in patients with ischemic stroke. Previous research also showed that elevated adiponectin was prospectively associated with MACCE and death in patients with type 2 diabetes and acute coronary syndrome [35], type 1 diabetes [36], type 2 diabetes [37], coronary artery disease [38] and community-based population [39]. A meta-analysis showed that increased plasma levels of adiponectin related to a high risk of mortality in subjects with CVD [40], while another study reported that high plasma adiponectin levels at discharge were associated with all-cause mortality during the 11.6 years follow-up in patients with acute myocardial infarction [41]. Sasso et al. [42] showed that adiponectin played a role in progression of any stage of ischemic heart disease also in normal glucose tolerance subjects. However, Zoccali et al. [43] found that high plasma adiponectin levels related to a low risk of cardiovascular outcomes in patients with end-stage renal disease, suggesting this protein was as a protective cytokine for the cardiovascular system. In addition, the correlation between adiponectin and further cardiovascular events in type 2 diabetes had not been confirmed in another study [44]. In addition, a meta-analysis suggested that association between adiponectin levels and risk of coronary heart disease was comparatively moderate  [45]. Furthermore, Laughlin et al. [46] did not support use of adiponectin for CVD risk stratification.

A meta-analysis suggested that the paradoxical association between adiponectin and mortality might be modulated by BNP [47]. Drechsler et al. [48] showed that increased adiponectin during follow-up related to a higher risk of adverse cardiovascular outcomes and death in hemodialysis patients and this association could be weakened by increased levels of NT-pro-BNP. Similarity, another study suggested that the apparently positive relationship between adiponectin and risk of CVD and mortality in asymptomatic elderly men might be achieved by elevated NT-pro-BNP [49]. Furthermore, Masuch et al. [50] found that elevated NT-proBNP might be associated with adiponectin signaling in cardiac healthy individuals. Consistent with those findings, we also found that the predictive value of adiponectin was more pronounced in patients with high levels of NT-pro-BNP (P < 0.001).

Menzaghi et al. [51] showed that the association between adiponectin and cardiovascular mortality was observed in men but not women, suggesting a sex-specific manner [51]. A similar sexual dimorphism had been reported for the relationships between adiponectin chronic kidney disease [52] and type 2 diabetes [50]. In healthy middle-aged men, Prugger et al. [53] reported that elevated adiponectin plasma levels related to a high 10-year risk of ischemic stroke. In the largest community-based African American cohort, elevated adiponectin was associated among women not men with a higher risk of incident stroke [54]. However, our results showed that the predictive value of adiponectin was significant in both sexes and more pronounced in women.

Several drugs may affect adiponectin serum levels. In patients with type 2 diabetes, SGLT2 inhibitor treatment was associated with increased circulating adiponectin levels [55]. A meta-analysis showed a significant increase in adiponectin plasma levels after statin therapy (weighted mean difference: + 0.57 μg/mL) [56], suggesting a significant association with clinical implications [57]. Furthermore, another meta-analysis found that Sitagliptin and vildagliptin increased serum adiponectin levels  [58]. However, we did not obtain that information. The association between those drugs and adiponectin levels should be explored in future research.

The role of elevated adiponectin in stroke prognosis had not been elaborated. Adiponectin resistance and the confounding role of BNP had been proposed. First, several studies suggested that adiponectin resistance might lead to elevated levels of adiponectin, which would in turn predict high CVD mortality [59]. Second, BNP might directly increase adiponectin expression [60]. The correlation between adiponectin and mortality might be achieved through the BNP pathway [61]. However, in this study, adjusting NT-pro BNP did not change this prediction relationship. Third, reduced kidney function might act as a confounder of the association between adiponectin and mortality [8]. However, in this study, patients with renal insufficiency were excluded and eGFR were adjusted. Lastly, adiponectin could exacerbate inflammatory process in several tissues and cell types [8] and exerted proinflammatory effects by inducing chemokine production [62]. Adiponectin could increase systemic inflammation as a way to counteract proinflammatory conditions [27]. Similarly, a positive association between CRP and adiponectin had been proposed in this study.

## Strengths and limitations

Our study is the first analysis of the correlation between adiponectin and risk of MACCE (mortality) in ischemic stroke patients. Furthermore, a wide range of potentially confounding risk factors, such as sex, BMI, kidney function and serum levels of BNP were collected to adjust, allowing us to observe the independent effect of adiponectin.

Some limitations also need to be considered. First, we only included Chinese. The association between adiponectin and outcomes in other populations could not be confirmed. Second, we only tested blood levels of adiponectin and no information about cerebral spinal fluid (CSF) had been obtained. In fact, a positive correlation between the plasma and CSF levels of adiponectin had been found [63]. In addition, only a single time-point blood draw after overnight fast was available. Third, we only tested total adiponectin serum levels. In fact, three isoforms of adiponectin had been reported (high, middle, and low molecular weight) and high molecular weight adiponectin had the most biologically active [64]. Fourth, adiponectin exerts its effects through the activation of 3 receptors [5]. However, we did not test the activity of the adiponectin receptors. Furthermore, secretion of adipokines, including omentin-1 [65], retinol-binding protein 4 (RBP4) [66], fatty acid–binding protein 4 (FABP4) [67] was altered in adipose tissue dysfunction and might play role in the prognosis of stroke. A previous study also found that omentin plasma levels were inversely associated with intima-media thickness in diabetes patients with increased adiponectin levels [68]. However, in this study, we did not test those biomarkers, thus, the association between adiponectin, others adipokines and stoke outcomes could not be obtained. Lastly, an observational study could not draw any causal relationship. Moreno et al. [69] suggested that the paradoxical association between adiponectin and mortality was a cause-effect relationship.

## Conclusions

Elevated serum levels of adiponectin were associated with a higher risk of MACCE and mortality independent of traditional risk factors in ischemic stroke patients, indicating adiponectin play a role in progression of ischemic stroke.

## Data Availability

Please contact the correspondence author for the data request (Dr. Zeng).

## Abbreviations

HF: Heart failure
CVD: Cardiovascular disease
MACCE: Major adverse cardiovascular and cerebrovascular events
AIS: Acute ischemic stroke
NIHSS: National Institutes of Health Stroke Scale
DWI: Diffusion-weighted imaging
eGFR: Estimated glomerular filtration rate
CKD-EPI: Chronic Kidney Disease Epidemiology Collaboration
CRP: C-reactive protein
NT-proBNP: N-terminal of the prohormone B-type natriuretic peptide
IQR: Interquartile ranges
HR: Hazard ratio
CI: Confidence interval
ROC: Receiver operating characteristic
AUC: Area under the curve
NRI: Net reclassification improvement
